# Cerebrospinal fluid levels of opioid peptides in fibromyalgia and chronic low back pain

**DOI:** 10.1186/1471-2474-5-48

**Published:** 2004-12-09

**Authors:** James N Baraniuk, Gail Whalen, Jill Cunningham, Daniel J Clauw

**Affiliations:** 1 Center for the Advancement of Clinical Research, The University of Michigan, Ann Arbor, MI USA; 2 Chronic Pain and Fatigue Research Center, Division of Rheumatology, Immunology and Allergy, Room B107, Lower Level Kober-Cogan Building, Georgetown University, 3800 Reservoir Road, N.W. Washington, D.C. 20007-2197, USA

## Abstract

**Background:**

The mechanism(s) of nociceptive dysfunction and potential roles of opioid neurotransmitters are unresolved in the chronic pain syndromes of fibromyalgia and chronic low back pain.

**Methods:**

History and physical examinations, tender point examinations, and questionnaires were used to identify 14 fibromyalgia, 10 chronic low back pain and 6 normal control subjects. Lumbar punctures were performed. Met-enkephalin-Arg^6^-Phe^7 ^(MEAP) and nociceptin immunoreactive materials were measured in the cerebrospinal fluid by radioimmunoassays.

**Results:**

Fibromyalgia (117.6 pg/ml; 85.9 to 149.4; mean, 95% C.I.; p = 0.009) and low back pain (92.3 pg/ml; 56.9 to 127.7; p = 0.049) groups had significantly higher MEAP than the normal control group (35.7 pg/ml; 15.0 to 56.5). MEAP was inversely correlated to systemic pain thresholds. Nociceptin was not different between groups. Systemic Complaints questionnaire responses were significantly ranked as fibromyalgia > back pain > normal. SF-36 domains demonstrated severe disability for the low back pain group, intermediate results in fibromyalgia, and high function in the normal group.

**Conclusions:**

Fibromyalgia was distinguished by higher cerebrospinal fluid MEAP, systemic complaints, and manual tender points; intermediate SF-36 scores; and lower pain thresholds compared to the low back pain and normal groups. MEAP and systemic pain thresholds were inversely correlated in low back pain subjects. Central nervous system opioid dysfunction may contribute to pain in fibromyalgia.

## Background

Fibromyalgia (FM) is an enigmatic condition characterized by increased complaints of widespread pain with tenderness to palpation [1]. The tenderness is traditional tested by manually pressing over so-called tender points, but more recent studies have shown that the tenderness is generalized phenomenon [2]. The mechanisms responsible for the increase in the perception of pain in FM, and the variation of pain sensitivity in the general population are unclear. A similar continuum is seen with heat – induced pain. However, when subjects who report pain to a minimal stimulus (low pain threshold) were compared to subjects reporting less pain with the same stimulus (high pain threshold), there was enhanced functional magnetic resonance imaging (fMRI) responses in the low pain threshold group [3]. The differences in activation were greatest in the primary somatosensory cortex, anterior cingulate cortex and prefrontal cortex. These fMRI patterns suggest there may be a continuum within the population for pain thresholds, central cortical activation and verbalized pain perception. These results may be applicable to FM since the same brain regions have been identified in response to painful stimuli [4].

The pain present in FM may induce antinociceptive neural mechanisms with the release of opioid peptides. This hypothesis was tested by measuring opioid peptides in cerebrospinal fluid, and comparing these levels to systemic pain thresholds, subjective complaints, and quality of life measures in 3 sets of volunteers. FM and Chronic Fatigue Syndrome often overlap [5]. Chronic Fatigue Syndrome is characterized by severe fatigue associated with exertional exhaustion, pain symptoms, neurocognitive and sleep dysfunction [6]. Therefore, opioid levels were compared for FM and FM/ Chronic Fatigue Syndrome subsets. The second group had chronic low back pain (LBP) without FM or Chronic Fatigue Syndrome. These subjects have a chronic regional pain syndrome [7] and served as a positive control group. The negative control group was formed by healthy persons with no pain or fatigue.

Two peptides were selected for measurements because they were involved in antinociceptive responses. Alternatively, dysfunction of their release could predispose to chronic pain. Preproenkephalin A is the precursor for leucine-enkephalin, methionine-enkephalin (Met-enk), Met-enk-Arg^6^-Gly^7^-Leu^8^, and Met-enk-Arg^6^-Phe^7 ^(MEAP) [8]. MEAP was elevated in many brain regions in inflammatory models of arthritis and gluteal carregeenan injection in rats [9,10]. Nociceptin, also known as orphanin FQ, was increased in the cingulate gyrus in rat chronic pain models [9].

## Methods

### Subjects

FM, LBP and Normal control subjects between the ages of 18 and 70 years were recruited to this IRB-approved protocol from rheumatology and orthopedics clinics, advertisements, and word of mouth. Normal subjects were pain and chronic fatigue free, had no diabetic, neurologic, inflammatory, autoimmune, or other chronic disorder that could predispose to pain, alterations in sensation, or known variations in cerebrospinal fluid composition. LBP inclusion criteria were (i) dominant pain complaint of low back pain, and (ii) imaging studies within the past 6 months. Exclusion criteria were: (a) evidence of a lumbar fracture or tumor to explain the pain, (b) any chronic illness that may affect functional status such as diabetes, cancer, chronic obstructive pulmonary disease, chronic inflammatory diseases, renal insufficiency or similar debilitating disorders, (c) previous back or neck surgery, and (d) FM or Chronic Fatigue Syndrome. FM subjects had a prior clinical diagnosis of FM including widespread pain affecting all 4 quadrants and the axial skeleton lasting at least 3 months that was not explained by any other chronic illness, and the presence of at least 11 of 18 tender points when manual, digital pressure of ~4 kg was applied [1]. All subjects were medication-free for at least 4 days prior to study. Subjects participated in a 1/2 day protocol that involved confirmatory history and physical examination, questionnaires, tender point examinations, and lumbar puncture.

### Questionnaires

#### Systemic complaints questionnaire

This self – report questionnaire containing 44 queries grouped into the following modules: (i) Fatigue; (ii) Musculoskeletal: morning stiffness, muscle pain, muscle spasms, dry eyes, dry mouth, fingers sensitive to the cold, fingers turn blue and/or white in the cold, swollen lymph nodes, swollen joints, fever; (iii) Chest: shortness of breath (SOB), SOB when hurrying on level ground or walking up a slight hill, SOB when walking with other people of own age on level ground, stop for breath when walking at own pace on level ground, SOB when washing or dressing, rapid heart rate, chest pain, irregular heart rate, palpitations; (iv) Headaches: migraine or tension type; (v) Neurological: numbness or tingling of hands or legs, inability to concentrate, problems with memory, dizziness; (v) Ear, Nose and Throat (ENT): problems with balance, hearing loss, ear pain, sensation of ear blockage or fullness, ringing in the ears, sinus pain; (vi) Bladder: urinary urgency, pelvic discomfort / pain / pressure, persistent bladder fullness after urination, dysuria; and (vii) Irritable Bowel Syndrome (Rome I criteria): abdominal pain relieved with bowel movement, abdominal pain with a change in frequency or consistency of stool, changes in stool consistency, changes in stool form (hard or loose/watery), changes in passing of stool, bloating or feeling of abdominal distention, passage of mucus, nausea or vomiting [11]. Subjects were asked to respond "Yes" if they had recurrent or chronic symptoms for more than 3 of the past 12 months. The sum of positive responses for each module and the total were determined.

Subjects completed the MOS SF-36[12]. The domains were Physical Functioning (PF), Social Functioning (SF), Role Limitation due to Physical Problems (PP), Role Limitation due to Emotional Problems (EP), Mental Health (MH), Energy / Fatigue (E/F), Pain (P), General Perception of Health (GP) and Change in Health (CH).

### Pain threshold and tender point examinations

All subjects had pressure testing at 9 bilateral sites (18 total) using a hand held dolorimeter (algometer) with a 1 cm^2 ^rubber stopper making contact with the skin (Chatillon, etc.) [1,2]. The degree of pressure required to cause pain (pain threshold) was recorded at each site, and the number with pain induced by < 4 kg / cm^2 ^recorded. The average pressure causing pain was the Average Pain Threshold. FM and Normal subjects had manual digital pressure examinations of these points [1,2]. The number of points that were painful was recorded as Manual Tender Points.

### Cerebrospinal fluid (CSF) radioimmunoassays

Lumbar punctures were performed using local anesthetic and 23G spinal catheters. Volumes of 4 to 8 ml were obtained, centrifuged, aliquoted, and immediately frozen at -80°C. Samples were shipped on dry ice to Dr. Lars Terenius for measurement of neuropeptides. Neuropeptides were extracted from 1 ml aliquots using C-18 SepPak cartridges, eluted, dried, and resuspended for validated radioimmunoassays for MEAP [13,14] and nociceptin [15] using the standard methodologies developed in their laboratory. Concentrations in samples were interpolated from parallel standard curves. There was insufficient CSF to use HPLC for precise peptide identification. Hence, immunoreactive materials (irm) were measured as MEAP-irm and nociceptin-irm.

### Statistics

Geometric mean and 95% confidence intervals were determined for each neuropeptide, with arithmetic means and 95% CI's for all other variables. Differences between groups were assessed by ANOVA. Differences between means for each pair of groups were assessed by 2-tailed unpaired Student's t-tests with Bonferroni corrections for multiple comparisons. Significance was ascribed for p < 0.05.

## Results

### Demographics

Lumbar punctures were performed on 14 FM (1 male), 10 LBP (5 male) and 6 Normal (2 male) subjects. The averages and ranges of ages for these 3 groups were similar (overall average 42.7 yr, 38.8 to 46.6; 95% CI). There were 4 African-Americans in the FM group, and 1 each in the LBP and Normal groups, and 1 Asian in the FM group. The remainder was Caucasian.

### Systemic complaints questionnaire

**Figure 1:**
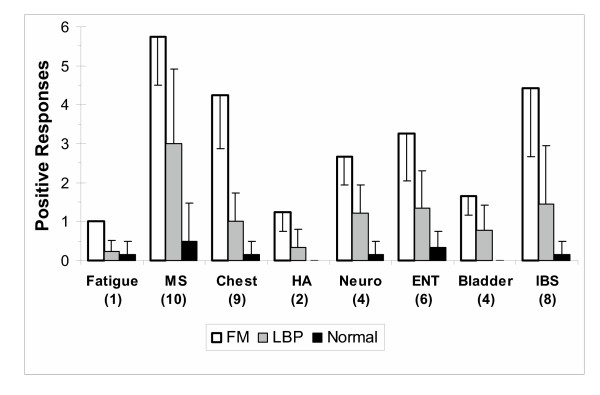
Systemic symptoms questionnaire results. FM (white bars), LBP (grey bars) and Normal (black bars) results are shown for each domain. The x-axis shows the systems domains with the total number of questions in parentheses. The y-axis shows the number of positive responses within each domain. The error bars are the 95% confidence intervals. FM scores were higher than Normal for ENT and IBS (p ≤ 0.05), Chest, Headache, Neurological and Bladder (p ≤ 0.01), Fatigue and Musculoskeletal (p ≤ 0.001). FM scores were higher than LBP for Neurological (p ≤ 0.05), Chest (p ≤ 0.01) and Fatigue (p ≤ 0.001). Normal and LBP scores were not different.

Significant differences were found between FM and Normal results by 2-tailed unpaired Student's t-tests with p < 0.05 after Bonferroni corrections for multiple comparisons of this data. All of the FM subjects complained of fatigue (figure 1). Other highly prevalent individual symptoms in FM were morning stiffness, muscle pain and spasms, and difficulties concentrating on cognitive tasks. FM scores were higher than Normal for ENT and IBS (p < 0.05), Chest, Headache, Neurological and Bladder (p < 0.01), Fatigue and Musculoskeletal (p < 0.001) complaints. When compared to LBP, FM scores were higher for Neurological (p < 0.05), Chest (p < 0.01) and Fatigue (p < 0.001) complaints. Normal and LBP scores were not different. Chronic Fatigue Syndrome co-existed with FM in 7 of the 14 subjects compared to none in the LBP and Normal groups. IBS was present in 58% of FM, 20% of LBP and 17% of Normal subjects.

### SF-36

**Figure 2:**
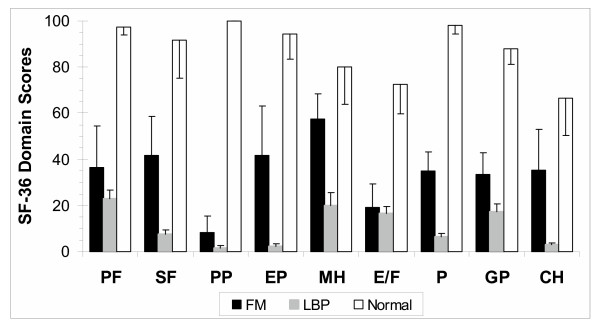
SF-36 domain results. Scores were ranked Normal (white bars with down-going 95%CI) > FM (black bars with up-going 95%CI) > LBP (grey bars with up-going 95%CI). The Normal group had significantly higher scores than FM for EP (p ≤ 0.05), SF (p ≤ 0.01), PF, PP, E/F, P and GP, but not MH or CH. All Normal means were highly significantly greater (p ≤ 0.001) than all LBP scores. FM scores were higher than LBP for EP, GP, and CH (p ≤ 0.05), SF (p ≤ 0.01), and MH and P (p ≤ 0.001). FM and LBP were not different for PF, PP or E/F.

Domain Scores for the Normal group were all near the predicted values of 100 (figure 2). FM scores were significantly lower for Emotional Problems (p < 0.05), Social Functioning (p < 0.01), Physical Functioning, Role Limitation due to Physical Problems, Energy / Fatigue, Pain and General Perception of Health (p < 0.001), but not Mental Health or Change in Health. All Normal means were highly significantly greater (p < 0.001) than all LBP scores. FM scores were higher than LBP for Emotional Problems, General Perception of Health, and Change in Health (p < 0.05), Social Functioning (p < 0.01), and Mental Health and Pain (p < 0.001). FM and LBP were not different for Physical Functioning, Role Limitation due to Physical Problems or Energy / Fatigue.

### Pain thresholds and tender point counts

**Figure 3:**
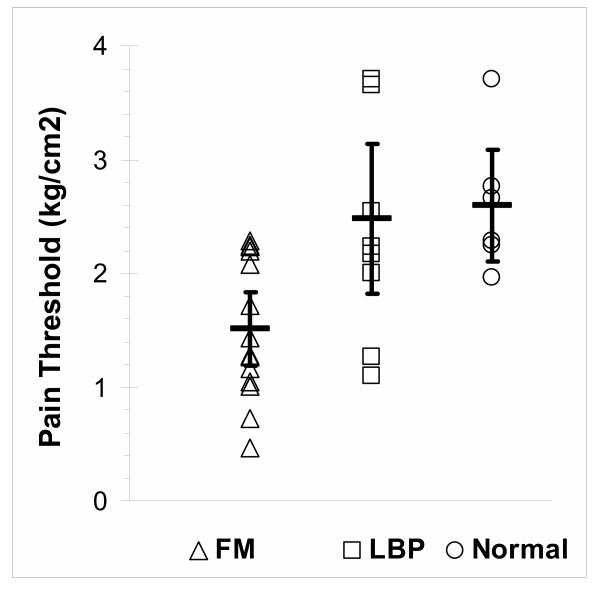
Pressure pain thresholds (kg/cm^2^) determined by dolorimetry. Pain thresholds for FM (triangles) were significantly lower than LBP (squares; p = 0.009) and Normal (circles; p = 0.002) groups. The bars indicated means and 95% confidence intervals.

**Figure 4:**
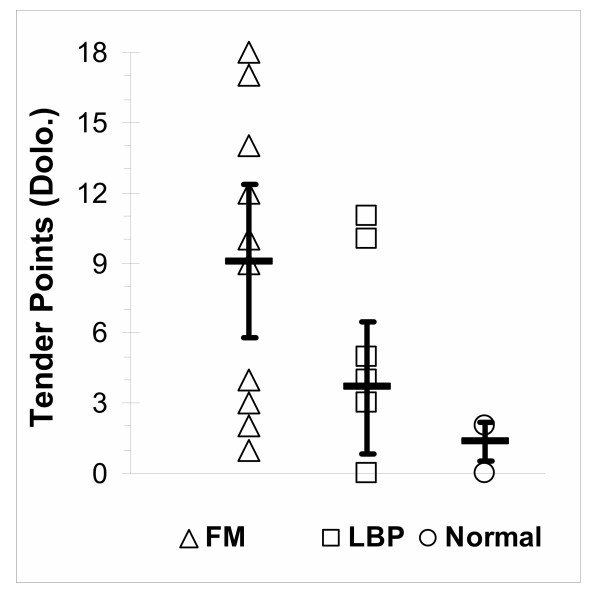
Tender points. The numbers of tender points detected by dolorimetry with ≤ 4 kg/cm^2 ^pressure were higher for FM (triangles, n = 14), than LBP (squares, n = 9, p = 0.03) and Normal (circles, n = 6, p = 0.008) groups. The bars indicated means and 95%CI's.

Dolorimetry identified significantly lower pressure pain thresholds for FM (1.51 kg/cm^2^) compared to the LBP (2.48 kg/cm^2^; p < 0.01) and Normal (2.60 kg/cm^2^; p < 0.001) groups (figure 3). The numbers of tender points determined by dolorimetry were 9.07 for FM, 3.67 for LBP, and 1.33 (p < 0.05 vs. FM) for Normal subjects (figure 4). Digital pressure identified more manual tender points in FM (13.00; 12.27 to 14.73) than Normal (4.67; 1.00 to 8.34; p < 0.001) groups. An average of 3.8 (1.90 to 5.70) more tender points were detected by manual examinations than by dolorimetry (p < 0.01). This difference has been attributed to higher anxiety and other psychometric variables in FM [16]. (Manual tender point counts were not recorded for LBP subjects.)

### Cerebrospinal fluid neuropeptide concentrations

#### MEAP – irm

**Figure 5:**
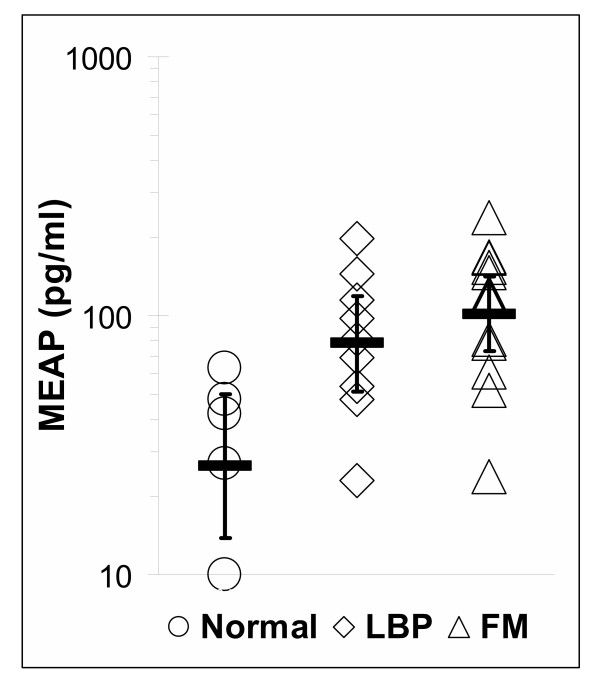
Met-Enkephalin-Arg^6^-Phe^7 ^(MEAP) concentrations (pg/ml) in cerebrospinal fluid (CSF) from normal (circles), low back pain (LBP, diamonds) and fibromyalgia (FM, triangles) subjects. The bars indicate geometric means and 95% confidence intervals. The groups were significantly different by ANOVA (p = 0.0014). MEAP in the FM (p < 0.01) and LBP (p < 0.05) groups were significantly higher than Normal (2-tailed unpaired Student's t-tests).

The 3 groups had significantly different mean CSF concentrations (p = 0.0014, ANOVA). The normal volunteers had significantly lower geometric mean concentrations (26.3 pg/ml; 13.9 to 49.9) than the FM (101.7 pg/ml; 72.8 to 142.0; p < 0.01 vs. normal), and LBP (78.0 pg/ml; 51.1 to 119.0; p < 0.05 vs. normal) (figure 5). Co-morbid Chronic Fatigue Syndrome did not affect the MEAP – irm results, since the arithmetic means were 112 pg/ml for FM subjects with this syndrome (n = 7) and 124 pg/ml with FM alone (n = 6). There were no obvious relationships with age, gender or race. The small sample size precluded further statistical analysis of these variables.

Nociceptin – irm was not different between FM (4.27 pg/ml, 3.22 to 5.66, n = 14), LBP (4.52 pg/ml, 3.12 to 6.55, n = 10) and Normal (5.65 pg/ml, 2.65 to 12.04, n = 6) groups.

### Systemic pain thresholds and MEAP – irm

**Figure 6:**
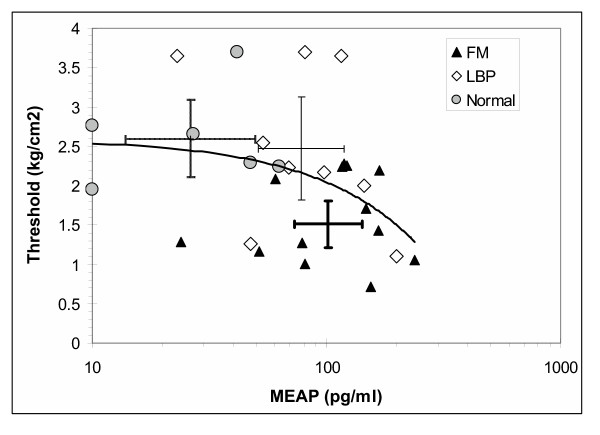
Semi-logarithmic relationships between pain thresholds and MEAP. FM subjects (black triangles) had pain thresholds below 2.3 kg/cm^2 ^(heavy, black error bars). Normal subjects (grey circles, cabled grey error bars) had lower MEAP and higher pain thresholds compared to FM. LBP subjects (open diamonds) had results that covered the full ranges for both variables (upper right error bars). The data for all subjects were linearly correlated (Pearson's correlation coefficient R = -0.38, p < 0.05) with an explained variance (R^2^) of 0.15. The line appears curved on this semi-logarithmic plot.

These 2 variables were correlated (Pearson's correlation coefficient of -0.38, p < 0.05; explained variance 0.15) when all subjects were examined as a single group (figure 6). This correlation was not found when the Normal and FM groups were examined by themselves. Normal subjects had higher thresholds and lower MEAP – irm concentrations. FM subjects had pain thresholds below 2.3 kg/cm^2^, which was coincidentally the lower 95% CI for the Normal group. MEAP – irm concentrations had a wide range in the FM subjects, but the geometric mean was significantly higher than for Normal subjects. Pain threshold and MEAP – irm concentrations did not have linear correlations in either the FM or Normal group. These data suggested that FM subjects were fundamentally different from Normal. When the pain threshold was below 2 to 2.3 kg/cm^2^, MEAP – irm levels increased approximately 4-fold compared to Normal. The combination of the widespread pain, systemic complaints, low pain threshold and high MEAP – irm concentrations in CSF was distinct from the low level of symptomatic complaints, normal (high) pain thresholds, and lower MEAP – irm levels found in the Normal group.

The LBP group had a chronic regional pain syndrome, no fibromyalgia, normal pain thresholds and systemic complaints, but severe disability (SF-36 scores). Only the LBP group showed a linear correlation between pain threshold and MEAP – irm concentrations (figure 6). The parameters of this correlation were similar to that of the entire group. This was due to the overlap of some high pain threshold / low MEAP – irm LBP subjects with the Normal group, and low pain threshold / high MEAP – irm LBP subjects with the FM data. This continuum of pain threshold and MEAP – irm levels in LBP was different from the clustered FM and Normal datasets, and suggested a different mechanism of MEAP – irm regulation in LBP from FM.

## Discussion

The Normal group had Systemic Complaints and SF-36 scores in the normal ranges, high pain thresholds, low numbers of manual and dolorimetry-derived tender points, and low CSF concentrations of MEAP – irm and nociceptin – irm.

The LBP group was a positive control for chronic regional pain. Their Systemic Complaints scores, systemic pain thresholds and dolorimetry defined tender point counts were not significantly different from Normal. However, most of their SF-36 results were near zero indicating the worst level of impairment of the 3 groups. They were the only group to show a correlation of decreasing pain thresholds with increasing MEAP – irm concentrations. The continuum of MEAP – irm levels in the LBP group led to borderline significance for the comparison to Normal levels. Inclusion of LBP subjects with higher or lower pain thresholds may have shifted the MEAP – irm concentration distribution towards or away, respectively, from the Normal group results. This is important when comparing these data to those of other studies. For example, chronic sciatica patients did not have elevated MEAP – irm compared to controls [33]. However, severity was not graded as extensively as in our study. Some subjects may have had less severe low back pain than in our group. If so, then the linear correlation noted in FIGURE 6 would predict no significant difference from normal subjects. Conversely, female LBP subjects with the lowest pain thresholds and highest MEAP – irm levels may have been making a transition from chronic low back pain to fibromyalgia [7]. Half of our LBP group was male, introducing gender as a potentially confounding factor.

The FM group's results were distinctly different from the Normal and LBP groups. FM had widespread pain complaints, the highest Systemic Complaints scores, the lowest pain thresholds, and highest numbers of tender points of the 3 groups. Their SF-36 scores were intermediate between LBP and Normal groups. Widespread pain, low pain thresholds, and high Systemic Complaints scores differentiated FM from Normal and LBP. CSF MEAP – irm concentrations were approximately 3-fold higher in FM than Normal (figure 5). This confirmed earlier findings [14] where a group of women meeting an older set of fibromyalgia criteria [17,18] had 34% higher CSF MEAP – irm concentrations than a group of 8 age-matched female control subjects [13]. None of the FM subjects in the earlier group required analgesics or other medications suggesting that their symptoms may have been milder than for our FM group. Our group contained 1 male and 13 females. In contrast, Lui et al. found MEAP concentrations (peptide identity confirmed by HPLC) that were 38% lower in FM than control subjects (p < 0.01) [19]. There was inadequate clinical data to compare the severity of complaints between these FM populations. These investigators also used a liquid-liquid peptide extraction method. The differences in FM severity, control groups, extraction procedures, and lack of sufficient CSF to identify precise peptides by HPLC [20] made it difficult to compare these sets of divergent results. Standardized measurements on CSF withdrawn from highly characterized and clearly defined subjects and controls will be required to resolve these inconsistencies.

This is the first investigation to examine the potential effect of co-existing CFS on MEAP – irm levels in FM. This suggested that the mechanism(s) of CFS were probably independent of those responsible for the elevated MEAP – irm levels in FM. Unfortunately, CFS subjects without FM could not be simultaneously tested to determine if their MEAP – irm levels were normal (as would be predicted).

Nociceptin – irm levels were the same in our three groups. The levels were about 10% of that found in women during labor [21]. It was unclear if the higher concentrations were due to pregnancy, neurohormonal adaptations during labor and delivery, or the effects of acute pain. Again, the absence of a control group comparable to our pain-free Normal group makes mechanistic comparisons difficult.

## Conclusions

The Normal group had Systemic Complaints and SF-36 scores in the normal ranges, high pain thresholds, low numbers of manual and dolorimetry-derived tender points and low cerebrospinal fluid MEAP and nociceptin concentrations. The LBP chronic regional pain group had similar Systemic Complaints scores, systemic pain thresholds and dolorimetry-defined tender point counts. However, most of their SF-36 results were near zero indicating the worst level of impairment of the 3 groups. MEAP – irm was just significantly elevated compared to the Normal group, and was correlated to the systemic pain threshold. The FM group was distinct since they had widespread pain complaints, the highest Systemic Complaints scores, the lowest pain thresholds, and highest numbers of tender points of the 3 groups. Their SF-36 scores were intermediate between the LBP and Normal groups. MEAP – irm concentrations were significantly higher in the FM than Normal group. The co-existence of Chronic Fatigue Syndrome with FM did not alter the MEAP – irm concentrations. This suggested that Chronic Fatigue Syndrome mechanism(s) did not involve preproenkephalin dysfunction. Nociceptin – irm levels were not different between these groups, and were lower than previously reported results from pregnant women in labor. Significant differences in MEAP – irm concentrations from previous studies may be due to the highly controlled definition of patients in this study, selection of control groups, and differences in peptide extraction methods.

## Abbreviations

CSF, cerebrospinal fluid; FM, fibromyalgia; fMRI, functional magnetic resonance imaging; irm, immunoreactive material; LBP, low back pain; MEAP, Met-enk-Arg^6^-Phe^7^; SF-36, short-form of 36 questions with the following domains: PF, physical functioning; SF social functioning; PP, role limitation due to physical problems; EP, role limitation due to emotional problems; MH, mental health; E/F, energy / fatigue; P, pain; GP, general perception of health; CH, change in health; Systemic Complaints domains: MS, musculoskeletal; HA, headache; Neuro, neurological; ENT, ear, nose & throat; IBS, irritable bowel syndrome (Rome I criteria); SOB, shortness of breath.

## Competing interests

The author(s) declare that they have no competing interests.

## Authors' contributions

JNB compiled the results and wrote the manuscript. DJC oversaw the clinical investigations that were performed by JC. GW was responsible for the sample repository, shipping, and collection of data. All authors read and approved the final manuscript.

## Pre-publication history

The pre-publication history for this paper can be accessed here:
